# Data for peptide-binding assay with oriented immobilization of GRP78 in Biacore

**DOI:** 10.1016/j.dib.2016.04.064

**Published:** 2016-05-04

**Authors:** Sheng-Hung Wang, John Yu

**Affiliations:** a Institute of Stem Cell and Translational Cancer Research, Chang Gung Memorial Hospital at Linkou, and Chang Gung University, Taoyuan 333, Taiwan

**Keywords:** SPR, surface plasmon resonance, NTA, nitrilotriacetic-acid, EDC, 1-Ethyl-3-(3-dimethylaminopropyl)-carbodiimide, NHS, N-Hydroxysuccinimide, Surface plasmon resonance, Biacore, GRP78, L-peptide, RLLDTNRPLLPY

## Abstract

To develop peptide-conjugated liposomes for cancer imaging and therapy, the label-free surface plasmon resonance (SPR) biosensor (Biacore™) is a practical and also preferred strategy to examine protein-peptide interaction. A new Biacore protocol with “oriented immobilization” for peptide-binding assay, which overcomes the drawbacks of conventional protocols, was presented in this data article. These results were complementary to the research article Wang at al., [1], which reported a series of new cancer-targeting peptides found with HotLig software (Wang et al., 2013) [2], and this newly developed Biacore protocol.

**Specifications Table**

**Table:** 

Subject area	*Biology*
More specific subject area	*Biochemical assays*
Type of data	*Figure*
How data was acquired	*Surface plasmon resonance (Biacore)*
Data format	*Analyzed*
Experimental factors	*Immobilization of His-tagged proteins on NTA sensor chip with amide linkages.*
Experimental features	*Peptide-binding assay using Biacore system with an oriented immobilization technique.*
Data source location	*Institute of Stem Cell and Translational Cancer Research, Chang Gung Memorial Hospital at Linkou, and Chang Gung University, Taoyuan, Taiwan*
Data accessibility	*Data are with this article*

## **Value of the data**

• The new Biacore strategy provided an alternative method which improved the stability of baseline and the sensitivity of peptide-binding assay.
• Immobilization of protein with defined orientation on sensor chip might be more favorable for analyzing molecular interactions.
• The slow dissociation rate from GRP78 implicated that the L-peptide and its analogs might be good candidates for drug-delivery of liposomes to cancer cells.

## 1. Data

**Fig. 1:**
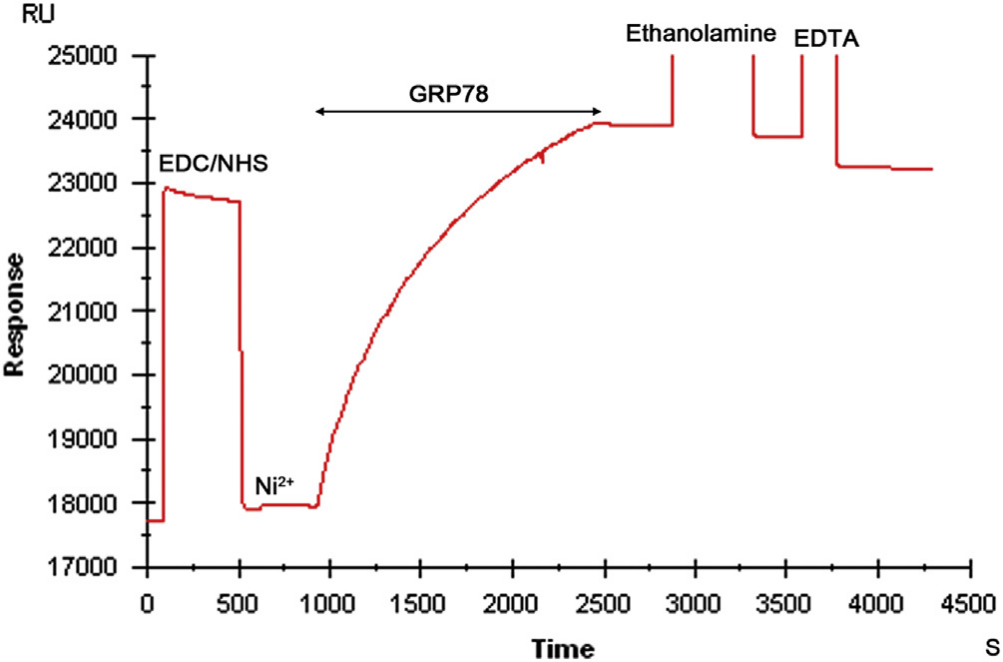
The nitrilotriacetic acid group on the chip NTA was activated by EDC/NHS for amine-coupling with GRP78 at neutral pH 7.4, while 6-His tag was conjugated to the N-terminus of GRP78 in order to chelate Ni^2+^ to NTA-chip surface, thereby orienting the C-terminal peptide-binding domain of GRP78 outwards (see also Fig. 4A in Ref. [1]). The resulting difference in resonance units (RU) for immobilized GRP78 was approximately 5515 RU as measured after de-activation with ethanolamine and washing with EDTA solution, indicating that GRP78 protein was covalently immobilized on chip NTA through amide linkage without Ni^2+^.

**Fig. 2:**
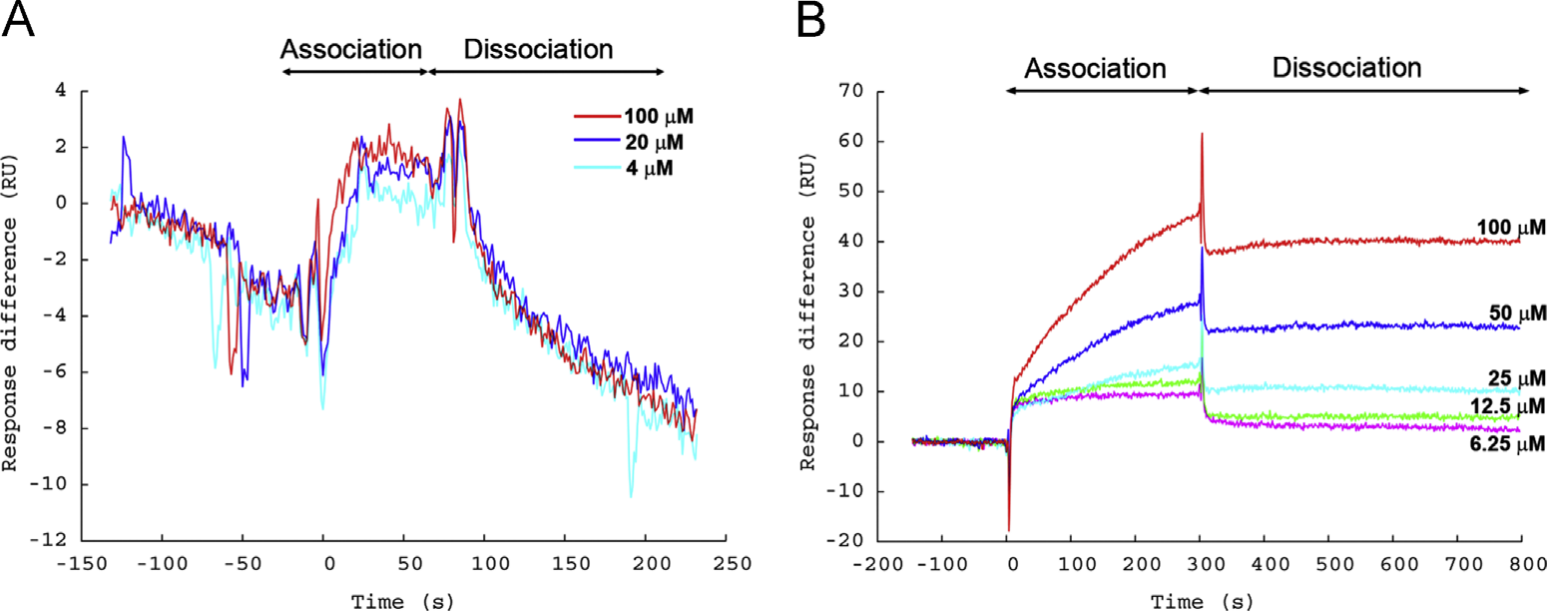
The baseline and sensitivity for detecting peptide binding were significantly improved by the modified Biacore protocol. (A) The sensorgram of Biacore assay for L-peptide binding to GRP78 using conventional protocol in which the protein was immobilized by Ni-chelation to chip NTA. The baseline declined continuously and the sensitivity for detection of peptide binding was poor, probably because of the loss of GRP78 from the chip. (B) Assay of L-peptide binding to human GRP78 using our modified protocol in which the protein was covalently linked to the chip NTA with a defined orientation. The baseline and sensitivity was stable and the response to peptide binding was dose-dependent. With the concentration of L-peptide ranging from 6.25 μM to 100 μM for the assay, L-peptide was observed to bind GRP78 with a slow dissociation rate. The *K*_D_ for L-peptide was approximately 10 μM, as estimated from fitting the binding curves using BIAevaluation version 4.1 (GE Healthcare) with default settings.

**Fig. 3:**
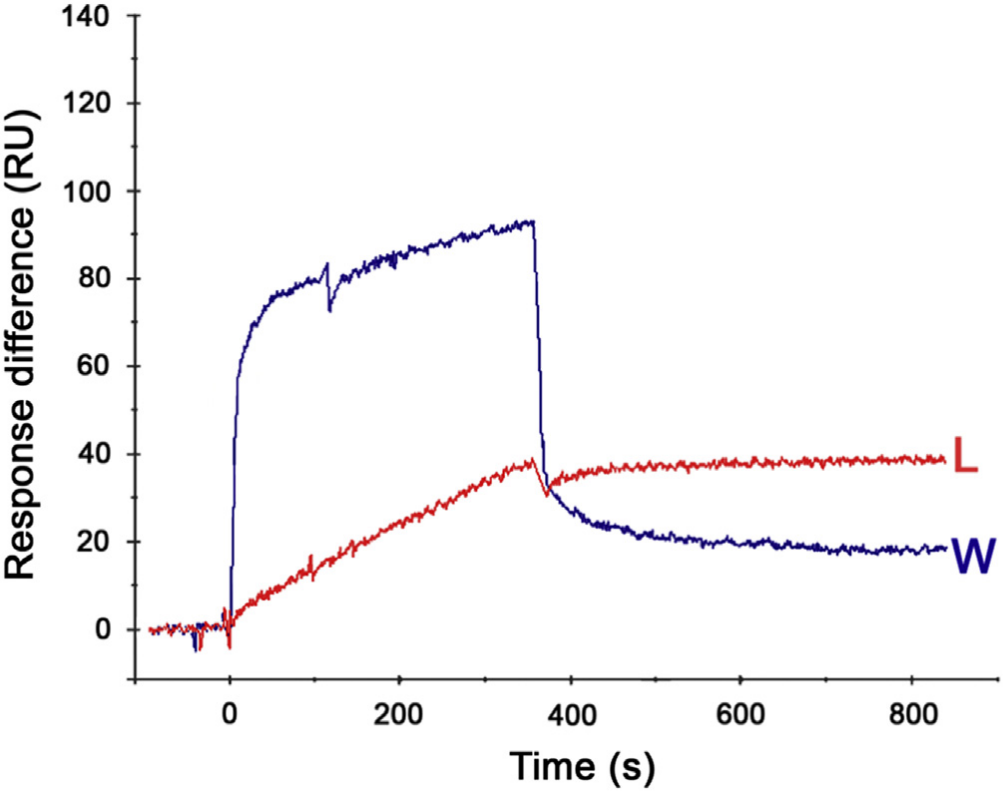
The binding of WIFPWIQL (W), a reported GRP78-binding peptide, was also examined. It was found that the W peptide dissociated from the GRP78 very fast. In contrast, the L-peptide (L) presented a slow rate of dissociation. Both peptides were evaluated at 50 μM. The analysis of molecular interactions with HotLig as described in Ref. [[1], [2]] indicated that there were seven hydrogen bonds between L-peptide and GRP78, whereas only one hydrogen bond was found to interact with W peptide.

This data article presents a Biacore protocol of “oriented immobilization of protein” on sensor chip (Fig. 1) for peptide-binding assay. The baseline and sensitivity for detecting peptide binding were significantly improved by this modified method (Fig. 2). The binding of two peptides, RLLDTNRPLLPY (L-peptide) [3] and WIFPWIQL (W) [4], to GRP78 were also compared using this new Biacore protocol (Fig. 3).

## 2 Experimental design, materials and methods

A surface plasmon resonance-based method using the Biacore X platform was developed to screen for peptides that targeted human GRP78. The sensor chip NTA, HBS-P buffer (pH 7.4), and the amine coupling kit (which includes EDC, NHS, and ethanolamine reagents) were all obtained from GE Healthcare. The sensor chip NTA was activated using EDC and NHS reagents, according to the standard procedure for amine coupling. A one-minute pulse of NiCl_2_ solution (500 μM) was subsequently used to saturate the chip surface. N-His-tagged GRP78 (about 5 μg protein in 100 μl HBS-P buffer) was then covalently immobilized to the chip surface NTA under HBS-P buffer. Generally, the resulting differences in resonance units (RU) were about 5000~6000 RU. After de-activation with ethanolamine and washing with EDTA solution (0.35 M, pH 8.3), the prepared sensor chip was applied to Biacore X for the binding assay. HBS-P buffer containing 1 mM glycine was used as running buffer. A one-minute pulse injection of a solution of 20-mM sodium hydroxide dissolved in running buffer was used to regenerate the chip surface.
